# A Review on Sample Size Determination for Cronbach’s Alpha Test: A Simple Guide for Researchers

**DOI:** 10.21315/mjms2018.25.6.9

**Published:** 2018-12-28

**Authors:** Mohamad Adam Bujang, Evi Diana Omar, Nur Akmal Baharum

**Affiliations:** 1 Clinical Research Centre, Sarawak General Hospital, Ministry of Health, Sarawak, Malaysia; 2 Clinical Research Centre, Serdang Hospital, Ministry of Health, Selangor, Malaysia; 3 National Clinical Research Centre, Ministry of Health, Kuala Lumpur, Malaysia

**Keywords:** Cronbach’s alpha, internal consistency, reliability, sample size

## Abstract

**Background:**

Reliability studies are commonly used in questionnaire development studies and questionnaire validation studies. This study reviews the sample size guideline for Cronbach’s alpha test.

**Methods:**

Manual sample size calculation using Microsoft Excel software and sample size tables were tabulated based on a single coefficient alpha and the comparison of two coefficients alpha.

**Results:**

For a single coefficient alpha test, the approach by assuming the Cronbach’s alpha coefficient equals to zero in the null hypothesis will yield a smaller sample size of less than 30 to achieve a minimum desired effect size of 0.7. However, setting the coefficient of Cronbach’s alpha larger than zero in the null hypothesis could be necessary and this will yield larger sample size. For comparison of two coefficients of Cronbach’s alpha, a larger sample size is needed when testing for smaller effect sizes.

**Conclusions:**

In the assessment of the internal consistency of an instrument, the present study proposed the Cronbach’s alpha’s coefficient to be set at 0.5 in the null hypothesis and hence larger sample size is needed. For comparison of two coefficients’ of Cronbach’s alpha, justification is needed whether testing for extremely low and extremely large effect sizes are scientifically necessary.

## Introduction

Cronbach’s alpha is a measure of the internal consistency or reliability between several items, measurements or ratings. In other words, it estimates how reliable are the responses of a questionnaire (or domain of a questionnaire), an instrumentation or rating evaluated by subjects which will indicate the stability of the tools. Alpha was developed by Cronbach (1), which was originally used to measure the reliability of a psychometric instrument. The value of Cronbach’s alpha ranges from zero to one with the higher values implying the items are measuring the same dimension. In contrary, if the Cronbach’s alpha value is low (near to 0), it means some or all of the items are not measuring the same dimension (2–3).

For example, the Depression Anxiety and Stress Scale (DASS-21) that is used to measure the magnitude of respondents’ stress level condition for over the past week, has seven items measured by using a four-point Likert scale (ranging from zero to three) (4). To ascertain whether the items are reliable in measuring the same dimension, a test for Cronbach’s alpha may be used. The Cronbach’s alpha test is usually applied to test the consistency and stability of the questionnaires which measure latent variables. Although Cronbach’s alpha test may be applied in situations other than questionnaire development or validation, there is limited literature of its application in such scenarios.

Cronbach’s alpha has been applied in research to develop clinical tool. For instance, a study by Berg et al. (5) developed an instrument to measure the balancing ability in elderly patients. The study used a scale consisting of 14 movements of patients such as sitting unsupported, change of position and etc. The movements were evaluated by five physical therapists and they were given a score from zero to four for each movement. The high degree of internal consistency in this study showed that the scale measures the same dimension

One of the common issues in inferential studies is to determine the sufficient sample size. The lack of knowledge in sample size determination and unfamiliarity with sample size softwares are usually the challenges encountered by researchers especially among those who do not have adequate knowledge in statistics. Sufficient sample size is needed so that research conducted can provide reliable and reproducible evidence that can detect the desired consistency or stability of an instrument (questionnaire). It is an important aspect in research to avoid lack of power of the test due to underestimation of sample size and also prevent the waste of resources due to overestimation of sample size (6).

The purpose of this present paper is to provide a simple guide for medical researchers to plan sample size estimation for their studies that involve Cronbach’s alpha test. The sample sizes are determined and presented in the form of tables to estimate the minimum number of samples needed to obtain the desired value of Cronbach’s alpha. Following that, the discussion of this paper emphasizes on the application of the formula and the sample size tables.

### Cronbach’s Alpha Test

Discussion on Cronbach’s alpha test in terms of concept, applications, statistical test and sample size determination have been widely discussed in the literatures (7–11). Some of the discussions related to sample size are summarised in Table 1 (6, 12–16). The present study emphasizes the review of sample size determination for Cronbach’s alpha and focuses on sample size determination introduced by Bonett (6, 12).

### Hypothesis Testing for a Single Cronbach’s Alpha Test

The null hypothesis is H_0_: CA0 = CA1 and the possible selection of alternative hypotheses are:

H_a_: CA0 ≠ CA1 (this option yields a two-tailed test)
H_a_: CA0 < CA1 (this option yields a one-tailed test)
H_a_: CA0 > CA1 (this option also yields a one-tailed test)

CA0 refers to the value of Cronbach’s alpha in null hypothesis and CA1 refers to the value of Cronbach’s alpha in alternative hypothesis. Most commonly CA0 is assumed and set at 0 in which smaller sample sizes are derived since the gap between CA0 and CA1 is larger. Setting up the CA0 as not equal to 0 is uncommon in research but it is very useful in testing and comparing the hypothesis of two different alpha coefficients (13).

### Hypothesis Testing for Comparison of Two Cronbach’s Alpha Test

The null hypothesis is H_0_: ρ*_a_* = ρ*_b_* and the possible selection of alternative hypotheses are:

H_a_: ρ*_a_* ≠ ρ*_b_* (this option yields a two-tailed test)
H_a_: ρ*_a_* < ρ*_b_* (this option yields a one-tailed test)
H_a_: ρ*_a_* > ρ*_b_* (this option also yields a one-tailed test)

ρ*_a_* refers to the value of Cronbach’s alpha in population “a” and ρ*_b_* refers to the value of Cronbach’s alpha in population “b”.

## Methods

### Sample Size Calculation Based on Formula by Bonett (6)

Sample size for Coefficient alpha or Cronbach’s alpha was calculated using Microsoft Excel. The formulation was from Bonett (6) based on formula given;

(i) n=[{(2kk-1)(Zα2+Zβ)2}/ln(δ˜)2]+2

where

(ii) $$\delta =\frac{1-\text{CA}0}{1-\text{CA}1}$$

Sample size was calculated based on power of 0.80 and 0.90 (Power = 1–*β*) while probability of type I error (*α*) was set at 0.05 at all time. There are options on whether to use two-tailed test or one-tailed test settings. In this paper, the more-commonly used two-tailed test was chosen with the aim to detect the difference between the two Cronbach’s alpha. There are three things that need to be considered when determining the sample size for Cronbach’s alpha test: the number of items or raters (*k*), the value of Cronbach’s alpha at null hypothesis (CA0) and the expected value of Cronbach’s alpha (CA1). The value for CA0 and CA1 could be any value ranges from −1 to 1, however CA1 should not be equal to CA0 (CA1 ≠ CA0).

In order to illustrate the above formula with an example; there are 15 items in a questionnaire of which the reliability of its measurements need to be measured (CA0 and CA1 are identified at 0.0 and 0.7, respectively). Power is set at 90% and the value of alpha at 0.05. The minimum sample size required based on formula (i) and (ii) is as shown below:

Calculations:

$$\begin{array}{l} \alpha =0.05\\ \beta =0.1\\ k=15\\ \text{CA}0=0.0\\ \text{CA}1=0.7\\ \delta =\frac{1-0.0}{1-0.7}=3.333\\ n=\left[\frac{\left\{\left(\frac{2(15)}{15-1}\right){\left(Z_{0.025}=1.96+Z_{0.1}=1.282\right)}^{2}\right\}}{\text{ln}{\left(3.333\right)}^{2}}\right]+2\\ n=17.53\approx 18 \end{array}$$

Therefore, the minimum sample size required for this case study is approximately 18 samples.

### Sample Size Calculation Based on Formula by Bonett (12)

Sample size for comparing two Coefficient alpha or Cronbach’s alpha was also calculated using Microsoft Excel. The formulation was from Bonett (12) based on formula given;

(iii) $$n=\left[\frac{2\left(\frac{k1}{k1-1}+\frac{k2}{k2-1}\right){\left(Z_{\alpha /2}+Z_{\beta }\right)}^{2}}{\text{ln}{\left(\tilde{\delta }\right)}^{2}}\right]+2$$

where

(iv) $$\delta =\frac{\left(1-\rho_{a}\right)}{\left(1-\rho_{b}\right)}$$

Sample size was calculated based on power of 0.80 and 0.90 (Power = 1 – *β*) while probability of type I error (*α*) was set at 0.05 at all time. There are options on whether to use two-tailed test or one-tailed test settings. In this paper, the more-commonly used two-tailed test was chosen with the aim to detect the difference between the two Cronbach’s alpha. There are four things that need to be considered when determining the sample size for Cronbach’s alpha test: the number of items or raters in group one (*k_1_*), the number of items or raters in group two (*k_2_*), the value of Cronbach’s alpha at null hypothesis or group one (*ρ_a_*) and the expected or group two value of Cronbach’s alpha (*ρ_b_*). The value of *k_1_* and *k_2_* are set to be similar and present as *k*. The value for *ρ_a_* and *ρ_b_* could be any value ranges from −1 to 1, however *ρ_b_* should not be equal to *ρ_a_* (*ρ_b_* ≠ *ρ_a_*). In order to illustrate the above formula with an example; there are ten items in a questionnaire in group 1 and 2 for which the reliability of its measurements need to be measured (*ρ_a_* and *ρ_b_* are identified at 0.3 and 0.7, respectively). Power is set at 90% and the value of alpha at 0.05. The minimum sample size required based on formula (iii) and (iv) is as shown below:

Calculations:

$$\begin{array}{l} \alpha =0.05\\ \beta =0.1\\ k=10\\ \rho_{a}=0.3\\ \rho_{b}=0.7\\ \delta =\frac{\left(1-0.3\right)}{\left(1-0.7\right)}=2.3\\ n=\left[\frac{2\left(\frac{10}{10-1}+\frac{10}{10-1}\right){\left(1.96+1.282\right)}^{2}}{ln{\left(2.3\right)}^{2}}\right]+2\\ n=67.049\approx 68 \end{array}$$

Therefore, the minimum sample size required for this case study is approximately 68 samples per group.

## Results

Based on pre-specified alpha, power and effect size, the minimum sample size requirements are shown in Table 2 to Table 3. The pre-specified alpha and power are usually known (0.05 and 80.0% or 90.0%, respectively) and thus, the minimum sample size required is mainly determined by the value of *k* and the differences of the coefficients in null hypothesis and alternative hypothesis. The minimum requirement for sample size remains relatively unchanged when the value of *k* increases. Larger sample size was observed when the differences between the coefficients in the null and alternative hypotheses were smaller.

For one coefficient of Cronbach’s alpha test, if the coefficient of Cronbach’s alpha equals to zero in the null hypothesis, it is possible to derive a smaller sample size of less than 30 to achieve a minimum desired effect size of 0.7 or more (Table 2). However, setting the coefficient of Cronbach’s alpha larger than zero (e.g., CA0 = 0.50) in the null hypothesis could be necessary in some cases and this will yield a larger sample size. (Table 2). For a comparison of two coefficients of Cronbach’s alpha in different groups, a larger sample size is needed to detect smaller effect size when the coefficients in the null hypothesis (*ρ_a_*) and alternative hypothesis (*ρ_b_*) are nearly about the same (Table 3).

## Discussions

For a single coefficient of Cronbach’s alpha test, the application of the sample size tables can be used into two situations, which are sample size determination when CA0 can be assumed equal to zero and sample size determination when CA0 is assumed to be more than zero.

### Sample Size Determination for a Single Coefficient of Cronbach’s Alpha Test when CA0 can be Assumed Equal to Zero

In the questionnaire reliability studies, the coefficient of Cronbach’s alpha in the null hypothesis (CA0) is always assumed to be equaled to zero, implying that researchers assumed that there is no internal consistency of the whole items or for a particular domain of a questionnaire. Then, the coefficient of Cronbach’s alpha in the alternative hypothesis (CA1) will assume that there is a magnitude of internal consistency (either low, moderate or strong) of the questionnaire or a particular domain of a questionnaire. The minimum sample size requirement depends on the desired magnitude of internal consistency in the alternative hypothesis.

Higher differences of Cronbach’s alpha coefficients in the null hypothesis and alternative hypothesis will yield the lower sample size. Based on Table 2, in order to achieve an acceptable Cronbach’s alpha of 0.7, when the values of alpha and power are fixed at 0.05 and 80.0%, respectively, the sample size ranges from four (*k* = 100 and CA0 = 0.0 versus CA1 = 0.95) to 52 (*k* = 3 and CA0 = 0.0 versus CA1 = 0.5), depending on the number of *k* (number of items). Hence, Cronbach’s alpha test is suitable for conducting pilot studies when the CA0 is usually assumed to be zero. In studies related to questionnaire development and validation, Cronbach’s alpha is a common indicator for internal consistency and has always been evaluated in the pilot studies (17–20).

Based on the calculation, it is possible that the sample size can be as small as four with assumption of a very high internal consistency such as 0.95 or more (*k* = 100 and CA0 = 0.0 versus CA1 = 0.95). Although researchers may target a high coefficient of Cronbach’s alpha, however, it is impractical to do so as there is a possibility that it may not be achieved in an actual study. It is more reasonable to target a desired Cronbach’s alpha between 0.7 to 0.8 with an acceptable sample size.

An example of a sample size statement is as followed: “The aim of the study is to evaluate the internal consistency of particular questionnaire “A”. Questionnaire “A” has 15 items with a five-point Likert scale for every item. The coefficient of Cronbach’s alpha in the null hypothesis and alternative hypothesis are assumed to be equal to 0.0 and 0.7, respectively. Based on alpha value fixed at 0.05, the minimum sample size requirement is 14 in order to achieve power of 80.0%. The calculation is based on the formula introduced by Bonett (6).” If the questionnaire has a different number of items, researchers will need to calculate the sample size based on the formula in Equation (i).

### Sample Size Determination for a Single Coefficient of Cronbach’s Alpha Test when CA0 is Assumed to be More than Zero

Hypothesis testing when CA0 > 0 is rare in literature. However, the concept of testing the null hypothesis with CA0 > 0 may be useful in cases when researchers set a high target of internal consistency of an instrument. Some statistical soft-wares do not provide statistical test to assess internal consistency when CA0 is assumed to be larger than zero. However, with the sample size calculation, researchers can calculate and determine the sufficient sample size to assess the internal consistency when CA0 is assumed to be larger than zero.

For example, when *k* = 3 and CA0 = 0.50, based on alpha < 0.05 and power of at least 80.0%, a minimum sample size of 31 is sufficient to detect CA1 at 0.80. A larger minimum sample size is needed (*n* = 93) to detect CA1 at 0.70 (Table 2). An example of a sample size statement is as followed: “The aim of the study is to determine whether a particular questionnaire “B” has high magnitude of internal inconsistency. Questionnaire “B” has 10 items with a 5-point Likert scale for every item. The null hypothesis has set the coefficient of Cronbach’s alpha is at 0.50 while in the alternative hypothesis, the coefficient of Cronbach’s alpha is set at 0.80. Based on alpha of 0.05, the minimum sample size requirement is 23 to be able to detect at least 80.0% power of the test. The calculation is based on the formula introduced by Bonett (6).”

The present article recommends testing hypothesis with CA0 = 0.5 is necessary to test whether an instrument has an excellent internal consistency. However, it is a choice by the researchers to set the value of CA0, including setting CA0 as equals to zero. But, solely targeting a large difference of Cronbach’s alphas between CA0 and CA1 to get a lower sample size is not recommended. Therefore, justification is needed whether testing such effect size is scientifically necessary.

### Sample Size Determination when to Compare Two Cronbach’s Alpha in Two Different Groups

Hypothesis testing to compare two Cronbach’s alphas in two different groups is also rare in literature. However, this hypothesis testing is useful if the researcher aims to compare two coefficients of Cronbach’s alphas in two different groups. Although some statistical softwares might not provide statistical test to assess such hypothesis, researchers can calculate and determine sufficient sample size for the planned study to compare two Cronbach’s alphas in two different groups.

For example, when *k* = 3 and *ρ_a_* = 0.50, based on alpha = 0.05 and power = 80.0%, a minimum sample size of 183 is sufficient to detect *ρ_b_* at 0.70. A lower minimum sample size is needed (*n* = 59) to detect *ρ_b_* at 0.80 (Table 2). An example of a sample size statement is as followed: “The aim of the study is to determine whether the internal consistency of particular questionnaire “B” in group “two” is higher than in group “one”. Questionnaire “B” has 15 items with a 5-point Likert scale for every item. The coefficient of Cronbach’s alpha in group “one” was reported at 0.70 while the coefficient of Cronbach’s alpha in group “two” is hypothesised to be at least 0.80. Based on alpha of 0.05, the minimum sample size requirement is 207 to be able to detect at least 80.0% power of the test. The calculation is based on the formula introduced by Bonett (12).”

## Conclusion

This article provides sample size tables and simple guides for sample size estimation for Cronbach’s alpha. This guideline is useful for researchers especially in medical field where the recommended tables will ease them in estimating sample size for reliability studies. For one coefficient of Cronbach’s alpha test, the assumption of “the CA0 equals to zero” will usually yield a smaller sample size, and that is appropriate for pilot studies. However, to ensure an instrument has an excellent internal consistency, testing the hypothesis with CA0 larger than zero (CA0 > 0), such as CA0 = 0.5 is recommended.

Besides that, the calculation of sample size based on comparing two Cronbach’s alpha values in two different groups is also presented. Justifications are needed to decide whether the testing for extremely low or extremely large effect sizes are scientifically necessary. Hopefully, this guideline could help the researchers in sample size estimation for their researches to assess the reliability of an instrument.

## Figures and Tables

**Table 1:** The summarise on previous publication related sample size estimation for Cronbach’s alpha

No	Title	Author	Remarks
1	Sample size requirements for testing and estimating coefficient alpha.	Bonett DG (2002)	The sample size formulas closely approximate the sample size requirements for an exact confidence interval or an exact test.
2	Sample size requirements for comparing two alpha reliability	Bonett DG (2003)	Introduce sample size determination for comparing two alpha coefficients.
3	Minimum sample size for Cronbach’s coefficient alpha: a Monte Carlo	Yurdugül H (2008)	This paper suggests the sample size of 30 is sufficient on condition that first (largest) eigenvalue obtained from Principal Component Analysis (PCA) of the sample data set is higher than 6.00. However, if that first eigenvalues are between 3.00 and 6.00, then required minimum sample size is 100.
4	Cronbach’s alpha reliability: Interval estimation, hypothesis testing, and sample size planning	Bonett DG, Wright T (2015)	The results of a simulation study demonstrated that the proposed method performed better than alternative methods.
5.	Statistical methods–scale reliability analysis with small samples, Birmingham City University, Centre for Academic Success	Samuels P (2015)	Provides guideline for reliability analysis considering small samples.
6.	The RCSI sample size handbook: a rough guide	Conroy R (2016)	This study summarized that sample size of 30 can measure reliability using Cronbach’s alpha considering the scale items have strong correlations.

**Table 2:** Sample size tables for Cronbach’s alpha test with various effect sizes, alpha = 0.05 and power are set to 80.0% (*n^a^*) and 90.0% (*n^b^*), respectively

*k*	CA0	CA1	*n^a^*	*n^b^*	*k*	CA0	CA1	*n^a^*	*n^b^*	*k*	CA0	CA1	*n^a^*	*n^b^*
3	0.00	0.50	52	68	5	0.00	0.50	43	57	10	0.00	0.50	39	51
		0.55	39	52			0.55	33	44			0.55	30	39
		0.65	24	31			0.65	20	26			0.65	18	24
		0.70	19	24			0.70	16	21			0.70	15	19
		0.75	15	19			0.75	13	16			0.75	12	15
		0.80	12	15			0.80	10	13			0.80	9	12
		0.85	9	11			0.85	8	10			0.85	7	9
		0.90	7	8			0.90	6	7			0.90	6	7
		0.95	5	6			0.95	5	5			0.95	4	5
	0.50	0.65	188	250		0.50	0.65	157	209		0.50	0.65	140	186
		0.70	93	123			0.70	78	103			0.70	69	92
		0.75	52	68			0.75	43	57			0.75	39	51
		0.80	31	40			0.80	26	34			0.80	23	30
		0.85	19	24			0.85	16	21			0.85	15	19
		0.90	12	15			0.90	10	13			0.90	9	12
		0.95	7	8			0.95	6	7			0.95	6	7
	0.55	0.65	375	502		0.55	0.65	313	418		0.55	0.65	279	372
		0.70	146	194			0.70	122	162			0.70	109	145
		0.75	71	94			0.75	59	79			0.75	53	70
		0.80	38	50			0.80	32	42			0.80	29	38
		0.85	22	29			0.85	19	24			0.85	17	22
		0.90	13	16			0.90	11	14			0.90	10	13
		0.95	7	9			0.95	7	8			0.95	6	7
	0.60	0.65	1,323	1,770		0.60	0.65	1,103	1,476		0.60	0.65	981	1,312
		0.70	287	383			0.70	240	320			0.70	213	285
		0.75	109	145			0.75	91	121			0.75	81	108
		0.80	52	68			0.80	43	57			0.80	39	51
		0.85	27	35			0.85	23	30			0.85	21	27
		0.90	15	19			0.90	13	16			0.90	12	15
		0.95	8	10			0.95	7	9			0.95	7	8
	0.65	0.70	993	1,329		0.65	0.70	828	1,108		0.65	0.70	737	985
		0.75	210	281			0.75	176	235			0.75	157	209
		0.80	78	103			0.80	65	86			0.80	58	77
		0.85	35	46			0.85	30	39			0.85	27	35
		0.90	18	23			0.90	15	19			0.90	14	17
		0.95	9	11			0.95	8	9			0.95	7	9
	0.70	0.75	711	951		0.70	0.75	593	793		0.70	0.75	527	705
		0.80	146	194			0.80	122	162			0.80	109	145
		0.85	52	68			0.85	43	57			0.85	39	51
		0.90	22	29			0.90	19	24			0.90	17	22
		0.95	10	12			0.95	9	11			0.95	8	10
	0.75	0.80	475	636		0.75	0.80	397	530		0.75	0.80	353	471
		0.85	93	123			0.85	78	103			0.85	69	92
		0.90	31	40			0.90	26	34			0.90	23	30
		0.95	12	15			0.95	10	13			0.95	9	12
	0.80	0.85	287	383		0.80	0.85	240	320		0.80	0.85	213	285
		0.90	52	68			0.90	43	57			0.90	39	51
		0.95	15	19			0.95	13	16			0.95	12	15
	0.85	0.90	146	194		0.85	0.90	122	162		0.85	0.90	109	145
		0.95	22	29			0.95	19	24			0.95	17	22
15	0.00	0.50	38	49	20	0.00	0.50	37	49	25	0.00	0.50	37	48
		0.55	29	38		0.00	0.55	28	37			0.55	28	37
		0.65	18	23			0.65	17	23			0.65	17	22
		0.70	14	18			0.70	14	18			0.70	14	18
		0.75	11	14			0.75	11	14			0.75	11	14
		0.80	9	11			0.80	9	11			0.80	9	11
		0.85	7	9			0.85	7	9			0.85	7	9
		0.90	6	7			0.90	6	7			0.90	6	7
		0.95	4	5			0.95	4	5			0.95	4	5
	0.50	0.65	135	179		0.50	0.65	132	176		0.50	0.65	131	175
		0.70	67	89			0.70	66	87			0.70	65	86
		0.75	38	49			0.75	37	49			0.75	37	48
		0.80	23	29			0.80	22	29			0.80	22	29
		0.85	14	18			0.85	14	18			0.85	14	18
		0.90	9	11			0.90	9	11			0.90	9	11
		0.95	6	7			0.95	6	7			0.95	6	7
	0.55	0.65	269	359		0.55	0.65	264	353		0.55	0.65	261	349
		0.70	105	139			0.70	103	137			0.70	102	136
		0.75	51	68			0.75	50	67			0.75	50	66
		0.80	28	37			0.80	28	36			0.80	27	36
		0.85	16	21			0.85	16	21			0.85	16	21
		0.90	10	12			0.90	10	12			0.90	10	12
		0.95	6	7			0.95	6	7			0.95	6	7
	0.60	0.65	946	1,265		0.60	0.65	929	1,243		0.60	0.65	920	1,230
		0.70	206	275			0.70	202	270			0.70	200	267
		0.75	79	104			0.75	77	103			0.75	77	102
		0.80	38	49			0.80	37	49			0.80	37	48
		0.85	20	26			0.85	20	25			0.85	19	25
		0.90	11	14			0.90	11	14			0.90	11	14
		0.95	6	8			0.95	6	8			0.95	6	8
	0.65	0.70	710	950		0.65	0.70	698	933		0.65	0.70	691	924
		0.75	151	201			0.75	148	198			0.75	147	196
		0.80	56	74			0.80	55	73			0.80	55	72
		0.85	26	34			0.85	26	33			0.85	25	33
		0.90	13	17			0.90	13	17			0.90	13	16
		0.95	7	8			0.95	7	8			0.95	7	8
	0.70	0.75	508	680		0.70	0.75	500	668		0.70	0.75	494	661
		0.80	105	139			0.80	103	137			0.80	102	136
		0.85	38	49			0.85	37	49			0.85	37	48
		0.90	16	21			0.90	16	21			0.90	16	21
		0.95	8	10			0.95	8	9			0.95	8	9
	0.75	0.80	340	455		0.75	0.80	334	447		0.75	0.80	331	442
		0.85	67	89			0.85	66	87			0.85	65	86
		0.90	23	29			0.90	22	29			0.90	22	29
		0.95	9	11			0.95	9	11			0.95	9	11
	0.80	0.85	206	275		0.80	0.85	202	270		0.80	0.85	200	267
		0.90	38	49			0.90	37	49			0.90	37	48
		0.95	11	14			0.95	11	14			0.95	11	14
	0.85	0.90	105	139		0.85	0.90	103	137		0.85	0.90	102	136
		0.95	16	21			0.95	16	21			0.95	16	21
30	0.00	0.50	36	48	35	0.00	0.50	36	48	40	0.00	0.50	36	47
		0.55	28	37			0.55	28	36			0.55	28	36
		0.65	17	22			0.65	17	22			0.65	17	22
		0.70	14	17			0.70	14	17			0.70	14	17
		0.75	11	14			0.75	11	14			0.75	11	14
		0.80	9	11			0.80	9	11			0.80	9	11
		0.85	7	9			0.85	7	9			0.85	7	8
		0.90	6	7			0.90	6	7			0.90	6	7
		0.95	4	5			0.95	4	5			0.95	4	5
	0.50	0.65	130	173		0.50	0.65	130	173		0.50	0.65	129	172
		0.70	65	86			0.70	64	85			0.70	64	85
		0.75	36	48			0.75	36	48			0.75	36	47
		0.80	22	28			0.80	22	28			0.80	22	28
		0.85	14	17			0.85	14	17			0.85	14	17
		0.90	9	11			0.90	9	11			0.90	9	11
		0.95	6	7			0.95	6	7			0.95	6	7
	0.55	0.65	260	347		0.55	0.65	258	345		0.55	0.65	257	344
		0.70	101	135			0.70	101	134			0.70	100	134
		0.75	50	65			0.75	49	65			0.75	49	65
		0.80	27	36			0.80	27	35			0.80	27	35
		0.85	16	21			0.85	16	20			0.85	16	20
		0.90	10	12			0.90	10	12			0.90	10	12
		0.95	6	7			0.95	6	7			0.95	6	7
	0.60	0.65	913	1,222		0.60	0.65	909	1,216		0.60	0.65	905	1,211
		0.70	199	265			0.70	198	264			0.70	197	263
		0.75	76	101			0.75	76	100			0.75	75	100
		0.80	36	48			0.80	36	48			0.80	36	47
		0.85	19	25			0.85	19	25			0.85	19	25
		0.90	11	14			0.90	11	14			0.90	11	14
		0.95	6	8			0.95	6	8			0.95	6	7
	0.65	0.70	686	917		0.65	0.70	683	913		0.65	0.70	680	910
		0.75	146	195			0.75	145	194			0.75	145	193
		0.80	54	72			0.80	54	72			0.80	54	71
		0.85	25	33			0.85	25	33			0.85	25	33
		0.90	13	16			0.90	13	16			0.90	13	16
		0.95	7	8			0.95	7	8			0.95	7	8
	0.70	0.75	491	656		0.70	0.75	489	653		0.70	0.75	487	651
		0.80	101	135			0.80	101	134			0.80	100	134
		0.85	36	48			0.85	36	48			0.85	36	47
		0.90	16	21			0.90	16	20			0.90	16	20
		0.95	8	9			0.95	8	9			0.95	8	9
	0.75	0.80	329	439		0.75	0.80	327	437		0.75	0.80	326	435
		0.85	65	86			0.85	64	85			0.85	64	85
		0.90	22	28			0.90	22	28			0.90	22	28
		0.95	9	11			0.95	9	11			0.95	9	11
	0.80	0.85	199	265		0.80	0.85	198	264		0.80	0.85	197	263
		0.90	36	48			0.90	36	48			0.90	36	47
		0.95	11	14			0.95	11	14			0.95	11	14
	0.85	0.90	101	135		0.85	0.90	101	134		0.85	0.90	100	134
		0.95	16	21			0.95	16	20			0.95	16	20
45	0.00	0.50	36	47	50	0.00	0.50	36	47	55	0.00	0.50	36	47
		0.55	28	36			0.55	28	36			0.55	28	36
		0.65	17	22			0.65	17	22			0.65	17	22
		0.70	14	17			0.70	14	17			0.70	14	17
		0.75	11	14			0.75	11	14			0.75	11	14
		0.80	9	11			0.80	9	11			0.80	9	11
		0.85	7	8			0.85	7	8			0.85	7	8
		0.90	6	7			0.90	6	7			0.90	6	7
		0.95	4	5			0.95	4	5			0.95	4	5
	0.50	0.65	129	171		0.50	0.65	128	171		0.50	0.65	128	171
		0.70	64	85			0.70	64	85			0.70	64	85
		0.75	36	47			0.75	36	47			0.75	36	47
		0.80	22	28			0.80	22	28			0.80	22	28
		0.85	14	17			0.85	14	17			0.85	14	17
		0.90	9	11			0.90	9	11			0.90	9	11
		0.95	6	7			0.95	6	7			0.95	6	7
	0.55	0.65	257	343		0.55	0.65	256	342		0.55	0.65	256	341
		0.70	100	133			0.70	100	133			0.70	100	133
		0.75	49	65			0.75	49	65			0.75	49	64
		0.80	27	35			0.80	27	35			0.80	27	35
		0.85	16	20			0.85	16	20			0.85	16	20
		0.90	10	12			0.90	10	12			0.90	10	12
		0.95	6	7			0.95	6	7			0.95	6	7
	0.60	0.65	903	1,208		0.60	0.65	901	1,205		0.60	0.65	899	1,203
		0.70	196	262			0.70	196	262			0.70	196	261
		0.75	75	100			0.75	75	100			0.75	75	99
		0.80	36	47			0.80	36	47			0.80	36	47
		0.85	19	25			0.85	19	25			0.85	19	25
		0.90	11	14			0.90	11	14			0.90	11	14
		0.95	6	7			0.95	6	7			0.95	6	7
	0.65	0.70	678	907		0.65	0.70	677	905		0.65	0.70	675	903
		0.75	144	192			0.75	144	192			0.75	144	192
		0.80	54	71			0.80	54	71			0.80	54	71
		0.85	25	32			0.85	25	32			0.85	25	32
		0.90	13	16			0.90	13	16			0.90	13	16
		0.95	7	8			0.95	7	8			0.95	7	8
	0.70	0.75	485	649		0.70	0.75	484	648		0.70	0.75	483	646
		0.80	100	133			0.80	100	133			0.80	100	133
		0.85	36	47			0.85	36	47			0.85	36	47
		0.90	16	20			0.90	16	20			0.90	16	20
		0.95	8	9			0.95	7	9			0.95	7	9
	0.75	0.80	325	434		0.75	0.80	324	433		0.75	0.80	324	432
		0.85	64	85			0.85	64	85			0.85	64	85
		0.90	22	28			0.90	22	28			0.90	22	28
		0.95	9	11			0.95	9	11			0.95	9	11
	0.80	0.85	196	262		0.80	0.85	196	262		0.80	0.85	196	261
		0.90	36	47			0.90	36	47			0.90	36	47
		0.95	11	14			0.95	11	14			0.95	11	14
	0.85	0.90	100	133		0.85	0.90	100	133		0.85	0.90	100	133
		0.95	16	20			0.95	16	20			0.95	16	20
60	0.00	0.50	36	47	65	0.00	0.50	36	47	70	0.00	0.50	36	47
		0.55	28	36			0.55	28	36			0.55	27	36
		0.65	17	22			0.65	17	22			0.65	17	22
		0.70	14	17			0.70	13	17			0.70	13	17
		0.75	11	14			0.75	11	14			0.75	11	14
		0.80	9	11			0.80	9	11			0.80	9	11
		0.85	7	8			0.85	7	8			0.85	7	8
		0.90	6	7			0.90	6	7			0.90	6	7
		0.95	4	5			0.95	4	5			0.95	4	5
	0.50	0.65	128	170		0.50	0.65	128	170		0.50	0.65	128	170
		0.70	64	84			0.70	64	84			0.70	64	84
		0.75	36	47			0.75	36	47			0.75	36	47
		0.80	22	28			0.80	21	28			0.80	21	28
		0.85	14	17			0.85	13	17			0.85	13	17
		0.90	9	11			0.90	9	11			0.90	9	11
		0.95	6	7			0.95	6	7			0.95	6	7
	0.55	0.65	255	341		0.55	0.65	255	340		0.55	0.65	255	340
		0.70	100	132			0.70	99	132			0.70	99	132
		0.75	49	64			0.75	49	64			0.75	49	64
		0.80	27	35			0.80	27	35			0.80	27	35
		0.85	16	20			0.85	16	20			0.85	16	20
		0.90	10	12			0.90	10	12			0.90	10	12
		0.95	6	7			0.95	6	7			0.95	6	7
	0.60	0.65	898	1,201		0.60	0.65	897	1,199		0.60	0.65	896	1,198
		0.70	195	261			0.70	195	260			0.70	195	260
		0.75	75	99			0.75	75	99			0.75	75	99
		0.80	36	47			0.80	36	47			0.80	36	47
		0.85	19	25			0.85	19	25			0.85	19	25
		0.90	11	14			0.90	11	14			0.90	11	14
		0.95	6	7			0.95	6	7			0.95	6	7
	0.65	0.70	674	902		0.65	0.70	673	901		0.65	0.70	673	900
		0.75	144	191			0.75	143	191			0.75	143	191
		0.80	53	71			0.80	53	71			0.80	53	71
		0.85	25	32			0.85	25	32			0.85	25	32
		0.90	13	16			0.90	13	16			0.90	13	16
		0.95	7	8			0.95	7	8			0.95	7	8
	0.70	0.75	483	645		0.70	0.75	482	645		0.70	0.75	482	644
		0.80	100	132			0.80	99	132			0.80	99	132
		0.85	36	47			0.85	36	47			0.85	36	47
		0.90	16	20			0.90	16	20			0.90	16	20
		0.95	7	9			0.95	7	9			0.95	7	9
	0.75	0.80	323	432		0.75	0.80	323	431		0.75	0.80	322	431
		0.85	64	84			0.85	64	84			0.85	64	84
		0.90	22	28			0.90	21	28			0.90	21	28
		0.95	9	11			0.95	9	11			0.95	9	11
	0.80	0.85	195	261		0.80	0.85	195	260		0.80	0.85	195	260
		0.90	36	47			0.90	36	47			0.90	36	47
		0.95	11	14			0.95	11	14			0.95	11	14
	0.85	0.90	100	132		0.85	0.90	99	132		0.85	0.90	99	132
		0.95	16	20			0.95	16	20			0.95	16	20
75	0.00	0.50	36	47	80	0.00	0.50	36	47	85	0.00	0.50	36	47
		0.55	27	36			0.55	27	36			0.55	27	36
		0.65	17	22			0.65	17	22			0.65	17	22
		0.70	13	17			0.70	13	17			0.70	13	17
		0.75	11	14			0.75	11	14			0.75	11	14
		0.80	9	11			0.80	9	11			0.80	9	11
		0.85	7	8			0.85	7	8			0.85	7	8
		0.90	6	7			0.90	5	7			0.90	5	7
		0.95	4	5			0.95	4	5			0.95	4	5
	0.50	0.65	128	170		0.50	0.65	127	170		0.50	0.65	127	170
		0.70	63	84			0.70	63	84			0.70	63	84
		0.75	36	47			0.75	36	47			0.75	36	47
		0.80	21	28			0.80	21	28			0.80	21	28
		0.85	13	17			0.85	13	17			0.85	13	17
		0.90	9	11			0.90	9	11			0.90	9	11
		0.95	6	7			0.95	5	7			0.95	5	7
	0.55	0.65	254	340		0.55	0.65	254	339		0.55	0.65	254	339
		0.70	99	132			0.70	99	132			0.70	99	132
		0.75	49	64			0.75	49	64			0.75	48	64
		0.80	27	35			0.80	27	35			0.80	27	35
		0.85	16	20			0.85	16	20			0.85	16	20
		0.90	10	12			0.90	10	12			0.90	10	12
		0.95	6	7			0.95	6	7			0.95	6	7
	0.60	0.65	895	1,197		0.60	0.65	894	1,196		0.60	0.65	893	1,195
		0.70	195	260			0.70	195	260			0.70	194	259
		0.75	75	99			0.75	74	99			0.75	74	99
		0.80	36	47			0.80	36	47			0.80	36	47
		0.85	19	25			0.85	19	25			0.85	19	25
		0.90	11	14			0.90	11	14			0.90	11	14
		0.95	6	7			0.95	6	7			0.95	6	7
	0.65	0.70	672	899		0.65	0.70	671	898		0.65	0.70	671	897
		0.75	143	191			0.75	143	190			0.75	143	190
		0.80	53	71			0.80	53	70			0.80	53	70
		0.85	25	32			0.85	25	32			0.85	25	32
		0.90	13	16			0.90	13	16			0.90	13	16
		0.95	7	8			0.95	7	8			0.95	7	8
	0.70	0.75	481	643		0.70	0.75	481	643		0.70	0.75	480	642
		0.80	99	132			0.80	99	132			0.80	99	132
		0.85	36	47			0.85	36	47			0.85	36	47
		0.90	16	20			0.90	16	20			0.90	16	20
		0.95	7	9			0.95	7	9			0.95	7	9
	0.75	0.80	322	430		0.75	0.80	322	430		0.75	0.80	322	430
		0.85	63	84			0.85	63	84			0.85	63	84
		0.90	21	28			0.90	21	28			0.90	21	28
		0.95	9	11			0.95	9	11			0.95	9	11
	0.80	0.85	195	260		0.80	0.85	195	260		0.80	0.85	194	259
		0.90	36	47			0.90	36	47			0.90	36	47
		0.95	11	14			0.95	11	14			0.95	11	14
	0.85	0.90	99	132		0.85	0.90	99	132		0.85	0.90	99	132
		0.95	16	20			0.95	16	20			0.95	16	20
90	0.00	0.50	36	47	95	0.00	0.50	36	47	100	0.00	0.50	36	47
		0.55	27	36			0.55	27	36			0.55	27	36
		0.65	17	22			0.65	17	22			0.65	17	22
		0.70	13	17			0.70	13	17			0.70	13	17
		0.75	11	14			0.75	11	14			0.75	11	14
		0.80	9	11			0.80	9	11			0.80	9	11
		0.85	7	8			0.85	7	8			0.85	7	8
		0.90	5	7			0.90	5	7			0.90	5	7
		0.95	4	5			0.95	4	5			0.95	4	5
	0.50	0.65	127	170		0.50	0.65	127	169		0.50	0.65	127	169
		0.70	63	84			0.70	63	84			0.70	63	84
		0.75	36	47			0.75	36	47			0.75	36	47
		0.80	21	28			0.80	21	28			0.80	21	28
		0.85	13	17			0.85	13	17			0.85	13	17
		0.90	9	11			0.90	9	11			0.90	9	11
		0.95	5	7			0.95	5	7			0.95	5	7
	0.55	0.65	254	339		0.55	0.65	254	339		0.55	0.65	254	339
		0.70	99	132			0.70	99	132			0.70	99	132
		0.75	48	64			0.75	48	64			0.75	48	64
		0.80	27	35			0.80	27	35			0.80	27	35
		0.85	16	20			0.85	16	20			0.85	16	20
		0.90	10	12			0.90	10	12			0.90	10	12
		0.95	6	7			0.95	6	7			0.95	6	7
	0.60	0.65	893	1,194		0.60	0.65	892	1,194		0.60	0.65	892	1,193
		0.70	194	259			0.70	194	259			0.70	194	259
		0.75	74	99			0.75	74	99			0.75	74	99
		0.80	36	47			0.80	36	47			0.80	36	47
		0.85	19	25			0.85	19	25			0.85	19	25
		0.90	11	14			0.90	11	14			0.90	11	14
		0.95	6	7			0.95	6	7			0.95	6	7
	0.65	0.70	671	897		0.65	0.70	670	896		0.65	0.70	670	896
		0.75	143	190			0.75	143	190			0.75	143	190
		0.80	53	70			0.80	53	70			0.80	53	70
		0.85	25	32			0.85	25	32			0.85	25	32
		0.90	13	16			0.90	13	16			0.90	13	16
		0.95	7	8			0.95	7	8			0.95	7	8
	0.70	0.75	480	642		0.70	0.75	480	641		0.70	0.75	480	641
		0.80	99	132			0.80	99	132			0.80	99	132
		0.85	36	47			0.85	36	47			0.85	36	47
		0.90	16	20			0.90	16	20			0.90	16	20
		0.95	7	9			0.95	7	9			0.95	7	9
	0.75	0.80	321	429		0.75	0.80	321	429		0.75	0.80	321	429
		0.85	63	84			0.85	63	84			0.85	63	84
		0.90	21	28			0.90	21	28			0.90	21	28
		0.95	9	11			0.95	9	11			0.95	9	11
	0.80	0.85	194	259		0.80	0.85	194	259		0.80	0.85	194	259
		0.90	36	47			0.90	36	47			0.90	36	47
		0.95	11	14			0.95	11	14			0.95	11	14
	0.85	0.90	99	132		0.85	0.90	99	132		0.85	0.90	99	132
		0.95	16	20			0.95	16	20			0.95	16	20

**Table 3:** Sample size tables for comparisons of two Cronbach’s alpha test with various effect sizes, alpha = 0.05 and power are set to 80.0% (*n^a^*) and 90.0% (*n^b^*), respectively

*k*	*ρ^a^*	*ρ^b^*	*n^a^*	*n^b^*	*k*	*ρ^a^*	*ρ^b^*	*n^a^*	*n^b^*	*k*	*ρ^a^*	*ρ^b^*	*n^a^*	*n^b^*
2	0.5	0.6	1264	1691	8	0.5	0.6	723	967	30	0.5	0.6	655	876
		0.7	243	325			0.7	140	187			0.7	127	169
		0.8	77	103			0.8	45	60			0.8	41	54
		0.9	27	35			0.9	16	21			0.9	15	19
	0.7	0.8	384	514		0.7	0.8	221	295		0.7	0.8	200	267
		0.9	55	72			0.9	32	42			0.9	29	39
3	0.5	0.6	948	1269	9	0.5	0.6	712	952	40	0.5	0.6	649	868
		0.7	183	244			0.7	138	184			0.7	126	168
		0.8	59	78			0.8	45	59			0.8	41	54
		0.9	21	27			0.9	16	21			0.9	15	19
	0.7	0.8	289	386		0.7	0.8	217	290		0.7	0.8	198	265
		0.9	42	55			0.9	32	42			0.9	29	38
4	0.5	0.6	843	1128	10	0.5	0.6	703	940	50	0.5	0.6	646	864
		0.7	163	217			0.7	136	181			0.7	125	167
		0.8	52	69			0.8	44	58			0.8	41	54
		0.9	19	24			0.9	16	21			0.9	15	19
	0.7	0.8	257	343		0.7	0.8	215	287		0.7	0.8	197	263
		0.9	37	49			0.9	31	41			0.9	29	38
5	0.5	0.6	791	1058	15	0.5	0.6	678	907	60	0.5	0.6	644	861
		0.7	153	204			0.7	131	175			0.7	125	166
		0.8	49	65			0.8	43	56			0.8	41	53
		0.9	18	23			0.9	15	20			0.9	15	19
	0.7	0.8	241	322		0.7	0.8	207	276		0.7	0.8	197	262
		0.9	35	46			0.9	30	40			0.9	29	38
6	0.5	0.6	759	1015	20	0.5	0.6	666	891	70	0.5	0.6	642	859
		0.7	147	196			0.7	129	172			0.7	125	166
		0.8	47	63			0.8	42	55			0.8	40	53
		0.9	17	22			0.9	15	20			0.9	15	19
	0.7	0.8	232	309		0.7	0.8	204	272		0.7	0.8	196	262
		0.9	34	44			0.9	30	39			0.9	29	38
7	0.5	0.6	738	987	25	0.5	0.6	659	882	80	0.5	0.6	641	857
		0.7	143	190			0.7	128	170			0.7	124	166
		0.8	46	61			0.8	41	55			0.8	40	53
		0.9	17	21			0.9	15	19			0.9	15	19
	0.7	0.8	225	301		0.7	0.8	201	269		0.7	0.8	196	261
		0.9	33	43			0.9	30	39			0.9	29	38
